# A pilot study on biomarkers for tendinopathy: lower levels of serum TNF-α and other cytokines in females but not males with Achilles tendinopathy

**DOI:** 10.1186/s13102-016-0026-0

**Published:** 2016-02-25

**Authors:** James E. Gaida, Håkan Alfredson, Sture Forsgren, Jill L. Cook

**Affiliations:** University of Canberra Research Institute for Sport and Exercise (UCRISE), Canberra, Australia; Discipline of Physiotherapy, University of Canberra, ACT 2601 Canberra, Australia; Department of Surgical and Perioperative Sciences, Sports Medicine, Umeå University, Umeå, Sweden; Department of Integrative Medical Biology, Anatomy Section, Umeå University, Umeå, Sweden; Department of Community Medicine and Rehabilitation, Umeå University, S-901 87 Umeå, Sweden; Institute of Sport Exercise and Health, University College Hospital London, London, UK; La Trobe University Sport and Exercise Medicine Research Centre, Melbourne, Australia

**Keywords:** Cytokines, Tumor necrosis factor alpha, Biomarkers, Musculoskeletal pain

## Abstract

**Background:**

Achilles tendinopathy is a painful musculoskeletal condition that is common among athletes, and which limits training capacity and competitive performance. The lack of biomarkers for tendinopathy limits research into risk factors and also the evaluation of new treatments. Cytokines and growth factors involved in regulating the response of tendon cells to mechanical load have potential as biomarkers for tendinopathy.

**Methods:**

This case–control study compared serum concentration of cytokines and growth factors (TNF-α, IL-1β, bFGF, PDFG-BB, IFN-γ, VEGF) between individuals with chronic Achilles tendinopathy and controls. These were measured in fasting serum from 22 individuals with chronic Achilles tendinopathy and 10 healthy controls. Results were analysed in relation to gender and physical activity pattern.

**Results:**

TNF-α concentration was lower in the entire tendinopathy group compared with the entire control group; none of the other cytokines were significantly different. TNF-α levels were nevertheless highly correlated with the other cytokines measured, in most of the subgroups. Analysed by gender, TNF-α and PDGF-BB concentrations were lower in the female tendinopathy group but not the male tendinopathy group. A trend was seen for lower IL-1β in the female tendinopathy group. Physical activity was correlated with TNF-α, PDGF-BB and IL-1β to varying extents for control subgroups, but not for the female tendinopathy group. No correlations were seen with BMI or duration of symptoms.

**Conclusions:**

This pilot study indicates a lower level of TNF-α and PDGF-BB, and to some extent IL-1β among females, but not males, in the chronic phase of Achilles tendinopathy. It is suggested that future studies on tendinopathy biomarkers analyse male and female data separately. The lack of correlation between cytokine level and physical activity in the female tendinopathy group warrants further study.

## What is known about the subject

Tendinopathy has a lifetime incidence of more than 50 % in middle distance runners and presents a significant barrier to optimal performance in many elite sports. Much work has focussed on cytokine and growth factor expression at the tissue level, however, this approach is not suitable for longitudinal studies with multiple data collection points due to the trauma induced by the biopsy. Serum levels of cytokine expression overcomes this limitation but has received little research attention.

## What this study adds to existing knowledge

In this study, we found lower levels of TNF-α and PDGF-BB among females with chronic Achilles tendinopathy. Curiously, the association between physical activity and cytokine levels seen in the female control group was absent in the female tendinopathy group. These results should be seen as preliminary due to the low subject numbers, however, they provide direction and justification for further studies in this area.

## Clinical relevance

Potential biomarkers for chronic Achilles tendinopathy include TNF-α and PDGF-BB. These findings require verification in larger samples and should be extended to populations with recently developed tendinopathy.

## Background

Achilles tendinopathy is commonly seen in sports medicine and general practice. The cumulative lifetime incidence is 6 % in the non-athletic general population and 52 % in middle distance runners [1]. Contributing factors among athletes can include a sudden increase in loading [2, 3], a hard training surface [4], lower limb biomechanics [5] and calf muscle weakness [6]. Among non-athletic individuals, systemic factors such as diabetes [7] and blood lipids [8] may decrease the capacity of the tendon to tolerate everyday loads and contribute to tendinopathy in the absence of high tendon loading.

Serum biomarkers such as tumour necrosis factor alpha (TNF-α) are useful for monitoring disease severity and can even help to confirm a suspected diagnosis. For example, certain markers discriminate persons with bipolar disorder from controls [9]. In addition, an association between TNF-α and objective measures of depression and fatigue is shown in studies of chronic obstructive pulmonary disease (COPD) [10]. The TNF-α system is also strongly involved in inflammatory disorders such as inflammatory bowel disease and rheumatoid arthritis. Blood levels of both TNF-α and the soluble form of tumour necrosis factor receptor 1 (sTNFR1) [11] are increased among inflammatory bowel disease patients. sTNFR1 is generated when transmembrane TNFR1 is cleaved and the extracelluar receptor fragment is released [12]. At low concentrations, sTNFR1 stabilises the structure of TNF-α and slows its decay, while at higher concentrations it acts as a competitive antagonist for TNF-α action [13]. TNF receptor levels also correlate with disease activity for inflammatory bowel disease patients [14], and in rheumatoid arthritis TNF-α levels are increased in the synovial fluid of affected joints [15] and serum sTNFR1 levels are also elevated [16]. The presence of TNF-α and its two receptors has been confirmed in human Achilles tendon [17].

There is very little information on the effects of marked exercise on levels of TNF-α. In one study, in which the effects of heavy resistance exercise was evaluated for 30 resistance trained men, an initial increase but later, at recovery, a decrease in blood TNF-α levels was noted [18]. These authors also concluded that neuromuscular electrical stimulation appeared to prevent the decline in the circulating TNF-α that was observed during recovery [18]. In a study by Andersson and co-workers, it was found that the levels of TNF-α significantly increased after endurance physical exercise in non-athletes [19]. Likewise, it was found that there were increases in levels of TNF-α and other cytokines directly after 90-min soccer games in elite female players but that the levels were normalized within 21 h [20]. In a study on horses it was noted that the TNF-α blood level did not change in response to exercise competition but the authors concluded that circulating cytokines may after all be predictive of athletic performance [21]. In another study the mechanisms underlying overtraining syndrome for men was discussed and it was suggested that increased cytokine productions might be involved in this syndrome [22].

Only two human studies have investigated serum biomarkers for tendinopathy. In one study, serum levels of sTNFR1 and brain-derived neurotropic factor (BDNF) were no different between groups with and without tendinopathy [23]. However, gender-dependent correlations with physical activity levels were seen in the tendinopathy group but not in the control group. The other study investigated serum levels of oxidative derivatives of an omega-6 fatty acid (linoleic acid), which are known as oxylipins [24]. Other potentially interesting biomarkers for musculoskeletal diseases [25], such as tendinopathy, include TNF-α, interleukin 1 beta (IL-1β), basic fibroblast growth factor (bFGF), interferon gamma (IFN-γ), platelet derived growth factor BB (PDGF-BB) and vascular endothelial growth factor (VEGF).

It is a drawback that there is so little information concerning serum biomarkers in tendinopathy, as this condition is so frequently occurring among sports active individuals. Therefore, the aim of this study was to compare concentrations in serum of the cytokines referred to above between patients with Achilles tendinopathy and healthy controls. As the pattern of physical activity of patients with Achilles tendinopathy can influence biomarker concentrations [23], correlations with physical activity level were explored, as were correlations with two other factors [body mass index (BMI) and pain symptom length]. The hypothesis was that certain biomarker/s might show a special pattern for tendinopathy patients as compared with control persons.

## Methods

This project was approved by the Committee of Ethics at the Faculty of Medicine, Umeå University, and by the Regional Ethical Review Board in Umeå (04–157 M). All procedures were conducted according to the principles of the declaration of Helsinki. All participants gave written, informed consent.

Twenty-two individuals referred to the Sports Medicine Unit at Umeå University for management of chronic Achilles tendon pain were included in this study. All individuals had a diagnosis of mid-portion Achilles tendinopathy established by an experienced orthopaedic surgeon (HA). Diagnostic criteria included exercise-related pain in the Achilles tendon, localised 2 to 6 cm proximal to the calcaneal attachment. Only individuals with gradual onset of pain were included to avoid the possibility of including cases of partial rupture. The included patients had been referred to the Sports Medicine Unit following unsuccessful treatment of their condition with their local clinician (e.g. GP, physiotherapist). Previous treatments reported by these patients were rest from aggravating activities and heavy-load eccentric training. Ten control individuals with no history of Achilles tendon pain were recruited from the local community.

Both patients and controls had an ultrasound examination by the same orthopaedic surgeon (HA) using an Acuson Sequoia 512 (Siemens AG, Berlin, Germany) fitted with 8–13 MHz linear array transducer. From the prone position with both feet hanging off the end of the bed, the right and left Achilles tendons were examined using grey-scale and colour Doppler ultrasound. Characteristic features of tendinopathy were present in the patient group (increased anterior-posterior diameter, hypoechoic regions, and the loss of the clear demarcation between the anterior border of the tendon and Kager’s fat pad). Colour Doppler showed increased vascularity in the affected region of the painful tendon. All control participants had normal ultrasound examination findings.

Blood samples were collected in an identical manner for the tendinopathy and control groups. Blood was collected in the morning following an overnight fast in a serum separating tube, which was clotted at room temperature for 30 min and then centrifuged at 1300 × *g* for 10 min. Serum aliquots were stored at −80 °C awaiting analysis and did not undergo repeated freeze-thaw cycles.

Serum aliquots were thawed on ice immediately prior to analysis. Cytokine concentrations (TNF-α, IL-1β, bFGF, IFN-γ, PDGF-BB, and VEGF) were quantified using the Human Cytokine Group 1, 6-plex assay (catalogue number: X50007Z13H) on a Bio-Plex 200 Suspension Array System (Bio-Rad, Hercules, California, USA), according to the manufacturers instructions. Measurements were made in duplicate wells and all samples were tested on a single 96-well plate, which eliminated the possibility of plate-to-plate variation.

The limit of detection, defined as 2 standard deviations (SD) above the mean background fluorescence, is provided by the manufacturer based on measurement of 10 replicate wells containing all reagents except the cytokine of interest. The limit of detection for TNF-α is 3.0 pg/mL, for IL-1β 0.8 pg/mL, for bFGF 6.8 pg/mL, for IFN-γ 19.3 pg/mL, for PDGF-BB 1.0 pg/mL, and for VEGF 0.5 pg/mL.

The physical activity level of all participants was quantified using the Past Year Physical Activity Questionnaire (PYT-PAQ). The questionnaire prompts participants to estimate the frequency (months/year, days/week), duration (hours/day) and perceived intensity (sedentary, light, moderate, heavy) of occupational, household, and recreational activities over the past 12-months. These questionnaires are coded according to the typical metabolic cost of each activity to yield a metabolic equivalent (MET) of physical activity that is expressed in MET-hours/week.

The reproducibility of the MET-hours/week estimates from questionnaires completed 9-week apart is 0.70 (Spearman’s rank correlation coefficient) for occupational activity, 0.65 for household activitity, 0.73 for recreational activity and 0.64 for total activity [26]. This questionnaire has also been validated using 4 × 7-day physical activity diaries and 4 × 7-day accelerometer data, with the 4-time points in different weather seasons. Questionnaire validity against physical activity logs (intraclass correlation coefficient (ICC) = 0.42, 95 % CI = 0.28 to 0.54)) and against accelerometer data (ICC = 0.18 (95 % CI = 0.03 to 0.32) is comparable to other physical activity questionnaires.

Due to the relatively small and uneven group numbers, all analyses were conducted using non-parametric statistics. Continuous variables were tested using the Mann–Whitney *U* test. Significance level was set at *p* < 0.05 and Bonferroni adjustment was not used due to the pilot nature of this study.

The analysis was conducted first using all data points and then repeated following removal of any cytokine analysis result with a coefficient of variation (CV) greater than 15 %. Results are reported as mean (SD) throughout the manuscript unless otherwise indicated. Analysis was performed using STATA/IC 13.1.

## Results

Individuals with Achilles tendinopathy were well matched to the control participants according to key demographic variables (Table 1). There was a similar ratio of men to women, with 60 and 68 % of the patient and control group being male. Symptom data indicated that all patients had chronic tendinopathy as the shortest duration was 3.5 months.

**Table 1:** Participant demographics

	Achilles tendon status		
	Normal (*n* = 10)	Tendinopathy (*n* = 22)	*p*
Sex (male:female)	6:4	15:7	0.652
Age (years)	54.5 (8.4)	53.0 (10.8)	0.475
Height (cm)	172.1 (11.3)	175.2 (7.1)	0.416
Weight (kg)	82.5 (18.8)	86.9 (16.4)	0.490
BMI (kg/m^2^)	27.6 (3.8)	28.2 (4.2)	0.968
Physical activity (MET-hours/week)^a^	137.2 (29.1)	151.2 (54.2)	0.637
Pain: symptom duration (months)	N/A	24 (IQR 8 to 24) Range 3.5 to 120	

Data reported as mean (standard deviation (SD)) except symptom duration, which is given as median and interquartile range (IQR). ^a^Physical activity data missing for 1 individual in control group and 2 individuals in tendinopathy group

Serum concentration of TNF-α was significantly lower in individuals with Achilles tendinopathy, when females and males were grouped together, compared with the entire control group (Table 2). No differences were detected between the two groups for the other cytokines that were analysed. The findings were unchanged when the analysis was repeated after excluding data with CV >15 % (Table 3).

**Table 2:** Serum cytokine concentrations (all data points)

	Achilles tendon status		
Analyte (pg/mL)	Normal (*n* = 10)	Tendinopathy (*n* = 22)	*p*-value
TNF-α	173.58 (64.98)	114.40 (45.17)	0.018
IL-1β	13.08 (3.38)	11.18 (2.99)	0.193
bFGF	67.72 (26.60)	66.34 (26.53)	0.968
INF-γ	180.70 (74.90)	141.89 (46.94)	0.371
PDGF-BB	12041.49 (2813.68)	11062.68 (3447.73)	0.088
VEGF	203.21 (129.25)	217.05 (123.28)	0.626

**Table 3:** Serum cytokine concentrations (excluding observations with CV >15 %)

	Achilles tendon status				
Analyte (pg/mL)	n	Normal	n	Tendinopathy	*p*-value
TNF-α	8	179.52 (62.30)	18	119.93 (47.35)	0.030
IL-1β	10	13.08 (3.38)	22	11.18 (2.99)	0.193
bFGF	9	68.42 (28.11)	18	70.12 (27.89)	0.643
INF-γ	9	171.65 (73.43)	21	147.58 (39.57)	0.769
PDGF-BB	10	12041.49 (2813.68)	22	11062.68 (3447.73)	0.088
VEGF	9	211.36 (134.34)	20	232.60 (117.48)	0.5093

The mean CV for the cytokines analysis was 7.8 % (TNF-α), 5.3 % (IL-1β), 8.5 % (bFGF), 6.9 % (IFN-γ), 1.1 % (PDGF-BB), and 5.9 % (VEGF). Sixteen data points were removed due to a CV >15 %, which left 176 of the original 192 data points. As described above, repeating the analysis after removal of these data did not affect the results.

When separated by gender, serum TNF-α and PDGF-BB levels were lower in women with tendinopathy compared to women in the control group (Table 4). There was a non-significant trend for IL-1β to be lower in women with tendinopathy (*p* = 0.059). In contrast, no group differences were seen for men (*p* > 0.5 for all cytokines). After excluding data with CV >15 % there was a clear trend towards significance for a difference in TNF-α levels between women with and without tendinopathy (*p* = 0.053, Table 5). Significance testing for PDGF-BB (*p* = 0.023) and IL-1β (*p* = 0.059) levels between women with and without tendinopathy remained unchanged. The results for the men were unaffected by removal of data with CV >15 % (p > 0.3 for all cytokines).

**Table 4:** Serum cytokine concentrations analysed by sex and tendon status (all data points)

	Male			Female		
Analyte (pg/mL)	Normal (*n* = 6)	Tendinopathy (*n* = 15)	*p*	Normal (*n* = 4)	Tendinopathy (*n* = 7)	*p*
TNF-α	153.25 (65.81)	132.02 (30.06)	0.586	204.09 (58.13)	76.65 (50.93)	0.014
IL-1β	12.23 (3.79)	11.68 (2.08)	0.938	14.35 (2.60)	10.12 (4.37)	0.059
bFGF	76.64 (29.83)	72.54 (24.76)	0.815	54.35 (15.56)	53.049 (27.06)	0.850
INF-γ	168.99 (72.33)	150.93 (42.14)	0.876	198.26 (86.17)	122.53 (54.10)	0.257
PDGF-BB	11108.4 (3378.3)	11181.3 (2857.4)	0.640	13441.1 (613.2)	10808.5 (4737.1)	0.023
VEGF	238.97 (155.33)	240.2367 (130.653	0.876	149.57 (59.24)	167.4 (95.6)	0.571

**Table 5:** Serum cytokine concentrations analysed by sex and tendon status (excluding observations with CV >15 %)

	Male			Female		
Analyte (pg/mL)	Normal	Tendinopathy	*p*	Normal	Tendinopathy	*p*
TNF-α	167.65 (62.11)	135.77 (30.36)	0.301	199.29 (70.22)	78.72 (61.85)	0.053
	5	13		3	5	
IL-1β	12.23 (3.79)	11.68 (2.08)	0.938	14.35 (2.60)	10.12 (4.37)	0.059
	6	15		4	7	
bFGF	79.67 (32.30)	75.56 (25.30)	0.883	54.35 (15.56)	56.00 (2.26)	0.807
	5	13		4	5	
INF-γ	168.99 (72.33)	150.92 (42.14)	0.876	176.99 (91.78)	139.22 (34.25)	0.796
	6	15		3	6	
PDGF-BB	11108.4 (3378.3)	11181.3 (2857.4)	0.640	13441.1 (613.2)	10808.5 (4737.3)	0.023
	6	15		4	7	
VEGF	238.97 (155.33)	240.24 (130.65)	0.876	156.12 (70.7)	209.69 (70.28)	0.297
	6	15		4	5	

As TNF-α was different between the tendinopathy and control group, correlations with potential confounding factors (physical activity, BMI, pain symptom duration) were determined to further explore this finding (Table 6). A significant correlation was seen between TNF-α and physical activity level for all men, however, when examined by subgroup there was a correlation for the men of the control group but not those of the tendinopathy group. No other correlations were seen for physical activity, BMI or pain symptom duration.

**Table 6:** Spearman’s rank correlation between serum TNF-α and physical activity, BMI, and symptom duration

		Achilles tendon status (grouped by gender)					
		Total M	AT M	Control M	Total F	AT F	Control F
Physical Activity^a^	rho	0.5105	0.3846	0.9000	−0.2242	−0.6000	0.4000
	p	0.0255 19	0.1745	0.0374	0.5334	0.2080	0.6000
	n	19	14	5	10	6	4
BMI^b^	rho	−0.1338	−0.1607	−0.1429	0.2545	0.6071	0.2000
	p	0.5632	0.5672	0.7872	0.4500	0.1482	0.8000
	n	19	14	5	10	6	4
Pain: symptom duration^c^	rho		0.0335 14			−0.6773 7	
	p		0.9094			0.0946	
	n		14			7	

^a^Measured in MET-hours/week, ^b^measured in kg/m^2^, ^c^measured in months

Note: *M* male, *F* female, *AT* Achilles tendinopathy

Physical activity score was missing for 2 men and 1 woman, symptom duration was missing for 1 man with tendinopathy

Analysis of potential confounding factors was repeated for PDGF-BB (Table 7) and IL-1β (Table 8). There was no correlation between PDGF-BB and physical activity for all men, however, when examined by subgroup there was a negative correlation for the men of the tendinopathy group but not those of the control group. There was no correlation between PDGF-BB and physical activity for all women, however, when examined by subgroup there was a positive correlation for the women of the control group but not the tendinopathy group. There was no correlation between IL-1β and physical activity for all men, however, when examined by subgroup there was a positive correlation for the men of the control group but not the tendinopathy group. No correlations were seen for PDGF-BB or IL-1β with BMI or pain symptom duration.

**Table 7:** Spearman’s rank correlation between serum PDGF-BB and physical activity, BMI, and symptom duration

		Achilles tendon status (grouped by gender)					
		Total M	AT M	Control M	Total F	AT F	Control F
Physical Activity^a^	rho	−0.1895	−0.5868	0.7000	−0.0545	−0.4857	1.0000
	p	0.4372	0.0274	0.1881	0.8810	0.3287	0.0000
	n	19	14	5	10	6	4
BMI^b^	rho	−0.0831	−0.0536	−0.1429	0.2455	0.3214	0.0000
	p	0.7202	0.8496	0.7872	0.4669	0.4821	1.000
	n	21	15	6	11	7	4
Pain: symptom duration^c^	rho		0.2995			−0.4383	
	p		0.2982			0.3253	
	n		14			7	

^a^Measured in MET-hours/week, ^b^measured in kg/m^2^, ^c^measured in months

Note: *M* male, *F* female, *AT* Achilles tendinopathy

Physical activity score was missing for 2 men and 1 woman, symptom duration was missing for 1 man with tendinopathy

**Table 8:** Spearman’s rank correlation between serum IL-1β and physical activity, BMI, and symptom duration

		Achilles tendon status (grouped by gender)					
		Total M	AT M	Control M	Total F	AT F	Control F
Physical Activity^a^	rho	0.0018	−0.4154	0.9000	0.1152	−0.2571	0.8000
	p	0.9943	0.1397	0.0374	0.7514	0.6228	0.2000
	n	19	14	5	10	6	4
BMI^b^	rho	−0.0026	−0.0107	−0.1429	−0.0091	0.0357	0.4000
	p	0.9911	0.9698	0.7872	0.9788	0.9394	0.6000
	n	21	15	6	11	7	4
Pain: symptom duration^c^	rho		0.2704			−0.4383	
	p		0.3497			0.3253	
	n		14			7	

^a^Measured in MET-hours/week, ^b^measured in kg/m^2^, ^c^measured in months

Note: *M* male, *F* female, *AT* Achilles tendinopathy

Physical activity score was missing for 2 men and 1 woman, symptom duration was missing for 1 man with tendinopathy

A further question of interest was whether a systematic pattern of cytokine expression existed. Therefore, bivariate correlations between pairs of cytokines were examined using Spearmans rho (ρ) with TNF-α as the reference cytokine (Table 9). Strong correlations were observed between TNF-α and IFN-γ among all subgroups of men and women. Strong correlations were observed between TNF-α and bFGF, and between TNF-α and VEGF in men but not women. Finally, correlations between TNF-α and IL-1β, and between TNF-α and PDGF-BB were seen for all women but not when control and tendinopathy groups were examined separately. A limitation for this analysis was small group numbers.

**Table 9:** Spearman correlations between TNF-α and other cytokines

		Spearman’s correlations with TNF-α (grouped by gender and tendon status)					
Analyte (pg/mL)		Total M (*n* = 21)	AT M (*n* = 15)	Control M (*n* = 6)	Total F (*n* = 11)	AT F (*n* = 7)	Control F (*n* = 4)
TNF-α	rho	1	1	1	1	1	1
	p						
IL-1β	rho	0.5182	0.2321	1.0000	0.7182	0.3571	0.8000
	p	0.0161	0.4051	0.0000	0.0128	0.4316	0.2000
bFGF	rho	0.6442	0.5286	0.9429	0.2091	0.3214	0.2000
	p	0.0016	0.0428	0.0048	0.5372	0.4821	0.8000
INF-γ	rho	0.7067	0.6416	0.8857	0.7182	0.6786	1.0000
	p	0.0003	0.0099	0.0188	0.0128	0.0938	0.0000
PDGF-BB	rho	0.1312	−0.2536	0.7143	0.7182	0.4286	0.4000
	p	0.5709	0.3618	0.1108	0.0128	0.3374	0.6000
VEGF	rho	0.6844	0.5786	1.0000	0.1455	0.3929	0.4000
	p	0.0006	0.0238	0.0000	0.6696	0.3833	0.6000

## Discussion

This study of serum biomarkers for Achilles tendinopathy has three key findings. First, TNF-α levels were lower in women with tendinopathy than women without tendinopathy. Second, contrasting findings were seen for men and women across a number of outcomes. For example, PDGF levels were lower in women with tendinopathy but not in males with tendinopathy, and IL-1β showed a non-significant trend toward being lower in the female tendinopathy group as well. Third, the physical activity level correlated with TNF-α only for control men but not for any of the other groups. The finding for TNF-α was an unexpected finding as previous research mainly points toward an increased activation of the TNF-α system in tendinopathy and tendon overuse [17, 23, 27]. However, consistent with the findings in the present study, Pingel and collaborators [28] found decreased TNF-α mRNA levels in the most abnormal part of the affected tendon in comparison to 3 cm proximal in the same tendon. Together these data indicate that although the TNF-α system appears to be involved in tendinopathy, whether it is up or down regulated appears to depend on several factors. These factors appear to include whether measurement is made 1) at the mRNA or protein level, 2) in the tissue or blood, 3) of the cytokine itself, of its two receptors, or of the soluble receptor fragments. Importantly, it also appears as if the situation varies according to gender.

The influence of potential confounding factors on TNF-α levels was explored with correlation analysis in the various subgroups. The data showed a strong relationship between physical activity and TNF-α in the male control group. The same relationship was not seen in the male tendinopathy, the female control or female tendinopathy groups. It is well known that plasma TNF-α (and also sTNFR1 and sTNFR2) rises following strenuous and prolonged exercise (i.e. marathon running), and that the peak is seen during the first hour after the race [29]. In a study on the effects of 14-days of endurance physical activity (cross-country skiing) it was found that the levels of TNF-α increased significantly at week-1 and week-2 of the exercise but that they returned to baseline values during the recovery period [19]. The source of this increase is unclear, although it has been shown that neither blood mononuclear cells [30] nor skeletal muscle [31] is the source. It has been suggested that prolonged strenuous exercise causes gastrointestinal ischaemia, which leads to endotoxin leakage into the circulation and a secondary increase in TNF-α [32]. This is supported by evidence of no increase in serum or muscle TNF-α during prolonged but non-exhaustive exercise [33]. In contrast to exhaustive exercise, the transition to habitual aerobic exercise reduces plasma TNF-α in mildly overweight healthy women [34] whereas no effect is seen following 12-weeks of strength training in obese men [35] or obese hypertensive men and women [36], despite improvements in insulin sensitivity. Thus, the effect of exercise on serum TNF-α is dependent upon the intensity of exercise, whether it is a single bout or habitual and also to some extent on the obesity level of the participants. Of interest, several studies in this area note a relationship between plasma TNF-α and VO2max [37]. It is reasonable to draw a parallel between VO2max and physical activity (as measured in this study), as VO2max and the questionnaire used in this study have previously been shown to be moderately correlated (Spearman’s rank correlation = 0.37) [26]. However, it is unclear why the correlation between physical activity and TNF-α seen in control men was not detected in the tendinopathy group. It can be speculated that patients with tendinopathy experience pain with physical activities such as running and brisk walking, and therefore avoid these activities. The level of total physical activity did not differ between the tendinopathy and control group, however, the questionnaire used does not indicate the proportion of physical activity that is taken at high, moderate and low intensity. Notwithstanding this limitation, avoidance of painful activities might explain why there was the difference in TNF-α level between females with and without tendinopathy.

The association of potential confounders (physical activity, BMI, pain symptom duration) with PDGF-BB and IL-1β was also examined. Associations were only found for physical activity (tendinopathy males and control females for PDGF-BB, control males for IL-1β), but interestingly no associations were found for the most noteworthy group – females with tendinopathy. The explanation for this finding may be an interesting topic for future research.

In studies such as the one we conducted, it is important to clarify the potential influence of confounding factors. In contrast to physical activity (for control men), neither BMI nor duration of pain symptoms demonstrated a relationship with serum TNF-α, PDGF-BB or IL-1β. Therefore, in this study these do not appear to be important confounding factors. It is also worthwhile noting that the mean BMI of the participants was within the overweight category (25 – 30 kg/m^2^) but also that the two groups were well matched. Some previous studies in tendinopathy have shown that pain symptom duration is relevant, with one study finding that patellar tendinopathy biopsies showing VEGF protein expression were from individuals that had a shorter duration of symptoms than biopsies without VEGF expression [38]. In this case, neither BMI nor symptoms duration appear to be confounding factors for the cytokines and growth factors measured.

Very little is known regarding the role of TNF-α signalling in tendinopathy, necessitating a broader view across other research fields. The importance of the TNF-α system in chronic pain diseases has been recently shown in studies on humans and mice with arthritis; anti-TNF treatment leads to a rapid inhibition of pain responses in the central nervous system, with the effect occurring much more rapidly than the anti-inflammatory effect of TNF-α blockade [39]. In addition, it is known that preconditioning with a short ischaemic event reduces the severity of cerebral damage by a subsequent severe ischaemic event, and that this effect is induced by upregulation of sTNFR1 by TNF-α signalling [40]. Transient activity of the TNF-α system may result in a sustained increase in sTNFR1 expression [40]. Therefore, increased sTNFR1 expression, as is seen in Achilles tendon biopsies [17], may increase sensitivity to TNF-α stimulation as has been shown in other tissues [41]. Therefore, it remains of interest to examine TNF-α levels, as well as other cytokines, in the very early stages of tendinopathy, which will be the focus of future research endeavours.

A key finding was the contrast in results between men and women. This included finding lower levels of TNF-α (and a lower level in PDGF-BB and a trend for a decrease in IL-1β) in the female tendinopathy group, as compared to the control female group. Such a difference was not seen for males with tendinopathy compared to control males. Similarly, the correlation between TNF-α and physical activity that was seen in the male control group was not seen for the female control group. Finally, correlations between pairs of cytokines that were highly correlated for men (TNF-α & bFGF, TNF-α & VEFG) were not correlated among the female groups. This suggests that separate analyses for men and women are important for future research on tendinopathy biomarkers. This should be taken into consideration when doing future studies on levels of cytokines and closely related substances in relation to not only tendinopathy but also various sports related activities and complaints (see [42] for an overview of biomarkers in upper extremity musculoskeletal disorders).

There are several limitations of this study that should be acknowledged. First, this study had relatively small cohorts of tendinopathy patients and controls, and therefore may not have been adequately powered to detect changes in the cytokines studied. Second, this clinical cohort was heterogenous in terms of age, BMI and duration of symptoms, however, the groups means were actually well matched. Nevertheless, it is certainly possible that potential confounding factors not considered may have influenced the results. This might for example include menstrual cycle phases for women, which was not measured in this study. Third, cytokine levels were measured in serum and it is feasible that during processing, activated platelets affected the cytokine profile in the sample. It is also unknown to what extent serum levels of cytokines reflect intratendinous or paratendinous cytokine levels. While healthy tendons have a low vascular supply, there is a markedly increased vascular supply in chronic tendinopathy [43], and so the relationship between tendon and serum cytokine levels may be non-linear. However, all samples were taken at the same time of day and processed identically. Fourth, all the participants presented with chronic tendinopathy so at this stage we don’t know anything about serum cytokine levels in recently developed tendinopathy. Furthermore, the details concerning the physical activity pattern the days just before the day for the taking of the blood sample was not clarified; it was the general physical activity pattern that was taken into consideration. Identifying biomarkers of recently developed tendinopathy and in relation to the physical activity pattern just before the sample is taken are questions to be addressed in future research projects.

## Conclusion

This study identified lower serum TNF-α as a potential tendinopathy biomarker among females but not males. A lowering in PDGF-BB and a trend for a decrease in IL-1β was also seen in the female tendinopathy group, when compared to the female control group. A strong correlation between physical activity level and serum TNF-α was seen in the male control group but not the male tendinopathy group, nor in any of the female groups. Physical activity was also correlated with PDGF and IL-1β for certain subgroups. BMI and pain symptom duration were not associated with TNF-α, PDGF or IL-1β for any of the groups, and therefore do not appear to be confounding factors for these biomarkers in this study. The findings suggest that various cytokine systems, including the TNF-α system, are related to the presence of chronic Achilles tendinopathy. Further research on biomarkers of tendinopathy should analyse data for men and women separately and also extensively measure physical activity levels both short and long term.

## Abbreviations

BDNF: brain-derived neurotropic factor
bFGF: basic fibroblast growth factor
BMI: body mass index
COPD: chronic obstructive pulmonary disease
CV: coefficient of variation
ICC: intraclass correlation coefficient
IFN-γ: interferon gamma
IL-1β: interleukin 1 beta
MET: metabolic equivalent
PDGF-BB: platelet derived growth factor BB
PYT-PAQ: past year physical activity questionnaire
sTNFR2: soluble tumour necrosis factor receptor 2
sTNFR1: soluble tumour necrosis factor receptor 1
TNF-α: tumour necrosis factor alpha
VEGF: vascular endothelial growth factor
